# Study on In-Plane Initial Rotational Stiffness of Eccentric RHS Beam-Column Joints

**DOI:** 10.3390/ma16145103

**Published:** 2023-07-20

**Authors:** Xiaonong Guo, Weixin Li, Zeyu Xv

**Affiliations:** College of Civil Engineering, Tongji University, Shanghai 200092, China; guo-xiao-nong@tongji.edu.cn (X.G.); 2132342@tongji.edu.cn (W.L.)

**Keywords:** eccentric RHS joint, initial rotational stiffness, stiffener

## Abstract

The eccentric RHS (rectangular hollow sections) joint offers improved mechanical properties and better space utilization. Its use in frame structures has gained significant attention. Currently, the initial rotational stiffness of RHS joints, the simplified finite element analysis method of eccentric RHS joints, and the influence of the spatial effect of RHS joints are still unknown. The purpose of this research is to establish a calculation formula for the initial rotational stiffness of eccentric RHS joints, study the influence of the spatial effect under complex stress conditions, and propose a mathematical model that can be used to simplify the analysis of eccentric RHS joints. The research findings indicate that the web plate’s deformation stiffness primarily influences the joints’ initial rotational stiffness. This increases with a higher beam-to-column depth-to-width ratio, beam-to-column thickness ratio, and column width-to-thickness ratio. The form of the moment distribution in the joint changes, and begins to have a significant effect on the rotational stiffness when the beam-to-column flange width ratio reaches and exceeds 0.7. The stiffeners have a direct additive effect on the joint stiffness. The influence of adjacent beams on the joint is minimal, and the spatial effect of the joint can be disregarded. Furthermore, the finite element analysis confirmed the accuracy of the power function model in accurately simulating the static load behavior of the joint, particularly the bending moment–angle relationship.

## 1. Introduction

The application of steel tube structures can be traced back to the St. Louis Bridge in the United States in 1867 [1], marking the earliest use of such structures. In 1962, the Committee for International Development and Education on Construction of Tubular Structures (CIDECT) was established, focusing on research and development of offshore steel tube platform structures. The construction of the first offshore steel tube platform in the Gulf of Mexico in 1974 further popularized the use of steel tube structures, leading to the development of relevant design specifications [2]. Steel tube structures have wide applications in various public buildings such as stadiums, terminal buildings, and ocean platforms. Among the different types of joints used in steel tube structures, including bolted ball joints, welded ball joints, cast steel joints, flange joints, and intersecting joints, the intersecting joint stands out as one of the most representative forms. The intersecting joint is formed by directly welding the steel tube to the outer surface of the through rod through the end-intersecting line cutting. It offers advantages such as a simple structure, convenient construction, and direct force transmission.

In steel tube structures, the main components are tubular hollow sections and rectangular hollow sections. Consequently, intersecting joints are classified into tubular joints and RHS joints. Tubular joints have a longer research history compared to other types of intersecting joints. Scholars have extensively studied tubular joints with T-shaped [3,4], K-shaped [5], X-shaped [6], TT-shaped [7], and KK-shaped [8] configurations, resulting in mature research outcomes. The use of RHS joints emerged slightly later than that of tubular joints. RHS joints possess most of the advantages of tubular joints and offer the added convenience of easier processing of the rod end face. However, RHS joints exhibit an uneven stiffness distribution within the cross-sectional area, leading to more complex stress distribution at the joint connection. As a result, scholars have conducted numerous studies on the static performance of various types of RHS joints, yielding a wealth of research findings [9,10,11,12]. The research outcomes on intersecting joints have been incorporated into relevant design specifications [13,14,15,16].

The utilization of rectangular steel tubes as beam-column members and the adoption of eccentric intersecting joints in frame structures have gradually gained prominence in the construction of prefabricated buildings and outdoor elevator shafts. This approach allows for a smooth outer surface of the building, enhancing both the aesthetic appeal and space utilization. As part of this development, eccentric RHS joints have been introduced, wherein the axes of the beam-column members are perpendicular to each other, and the outer webs are flush. Notably, there are existing applications of eccentric RHS joints, such as the Shanghai World Expo Spain Pavilion [17]. Despite their practical application, there have been relatively few studies conducted on eccentric RHS joints. Zhao et al. [18,19] conducted a systematic analysis of the out-of-plane bearing capacity and stiffness of such joints. The research revealed that the primary failure mode of the joint is the buckling failure of the side wall of the main tube when the width of the main branch is relatively large. Conversely, when the width of the main branch tube is relatively small, the failure mode of the joint is characterized by plastic deformation of the main tube surface. Two formulas for calculating the ultimate bearing capacity have been derived based on these two failure modes.

Guo et al. conducted tests on eccentric RHS joints, and focused on summarizing the in-plane flexural capacity of eccentric RHS beam-column joints [20]. The findings revealed that the in-plane failure mode of these joints is a combination of column flange yielding and web buckling deformation. To further investigate the flexural bearing capacity of eccentric RHS joints under a certain axial force of the column member, 60 finite element models were established, and parametric analyses were performed. As a result, a calculation Formula (1) [20] for the flexural bearing capacity of eccentric RHS joints in the plane was derived.

(1) $$\Delta M_{u}=(0.397+0.345\cdot \beta^{*})\cdot f_{y}t_{l}hl$$

*M*_u_: Ultimate bending moment,

*t*_1_: Length of plate corresponding to stiffening,

*l*: Thickness of the plate corresponding to the stiffening,

*h*: Indicates the moment arm provided by the upper and lower stiffeners,

*f*_y_: The yield strength of the steel,

β*: Width ratio correction, which is related to the joint geometry.

Currently, the research on eccentric RHS joints still has the following problems: firstly, the research on eccentric RHS joints was limited to the bearing capacity. The research on the initial rotational stiffness of RHS joints is still lacking. Secondly, the finite element analysis of eccentric RHS joints was limited to solid elements. The simplified finite element analysis method of eccentric RHS joints is still lacking. Additionally, the research on eccentric RHS joints was limited to a single beam direction. In practical frame structure applications, a TT-shaped joint space with two adjacent beams at 90° is often formed, where both beams in different directions may experience significant loads. Therefore, the influence of the spatial effect under complex stress conditions is still unknown.

Based on the results of existing research [20], this study used ABAQUS to establish eccentric RHS joint models for verification and parameter influence analysis. The purposes of the research are as follows: firstly, to obtain the initial rotational stiffness of eccentric RHS joints by the height-to-width ratio (*η*), width ratio correction (β*), column diameter to thickness ratio (*γ*), beam to column wall thickness ratio (*τ*). The selection principles for these geometric parameters can be referenced from literature [21,22]. Secondly, this study analyzed the influence of the spatial effect under complex stress conditions. Finally, the research proposes a mathematical model that can be used to simplify the analysis of eccentric RHS joints.

## 2. Construction and Finite Element Model

### 2.1. Construction

**Figure 1:**
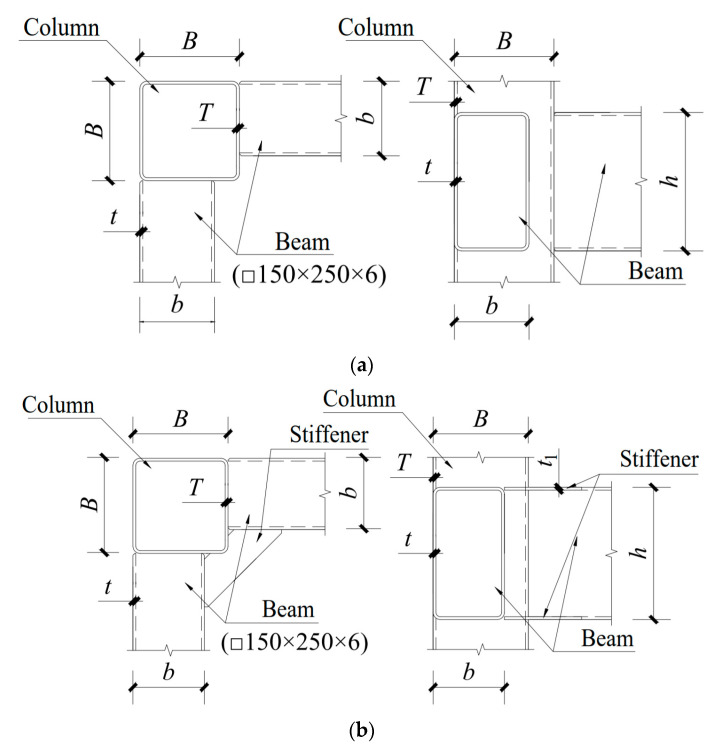
Models of joints. (**a**) Eccentric RHS joint without stiffeners; (**b**) Eccentric RHS joint with stiffeners.

The joint form studied in this paper is a T-shaped eccentric RHS joint. The joints are divided into two types: non-stiffener joints and stiffener joints. The beam and the outer edge of the column are aligned in a non-stiffener joint. A stiffener joint is based on the non-stiffener joint, and a stiffening thickness of 6 mm is welded on the upper and lower flanges of the beam and the column. The length of the stiffeners along the beam direction is 120 mm. The column member is a rectangular hollow section that is □ 200 × 8 mm, and the beam member is a rectangular hollow section of □ 250 × 150 × 6 mm. The joint constructions are shown in Figure 1.

### 2.2. Finite Element Model

**Figure 2:**
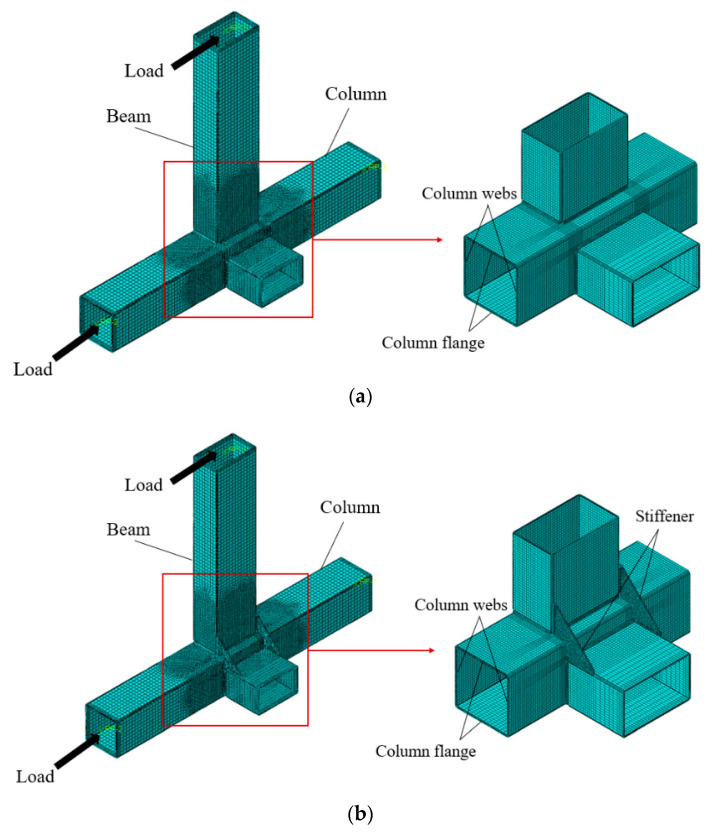
Meshes of finite element models. (**a**) Eccentric RHS joint without stiffeners; (**b**) Eccentric RHS joint with stiffeners.

The finite element software ABAQUS 2018 was used to simulate the eccentric RHS joints. Considering the calculation accuracy and costs comprehensively, all of the components, stiffeners, and welds in this research were divided into structured grids using a three-dimensional linear reduced integration element (C3D8R). The mesh of the finite element model not only needs to ensure the analysis accuracy of the calculation model, but also needs to consider the calculation efficiency. Therefore, the mesh of the finite element model established in this study was finer at the locations with complex stress distribution, large stress gradients, and complex contact relationships, and the mesh was relatively rough for the rest of the parts. The element size at the end of each member was 20 mm. The element sizes of 5 mm, 10 mm, and 20 mm were used for mesh refinement transitions from the intersecting surface to the rod end, respectively. It was divided into 4 layers in the thickness direction [23]. According to the above method, the mesh of the joint’s finite element model in this research is shown in Figure 2. The ultimate bending moments obtained from finite element analysis under different mesh sizes are presented in Table 1. The maximum mesh size of the finite element models in this research were set to be less than 90, in order to ensure the accuracy and efficiency of the calculations.

In the analysis of the eccentric RHS joints, a reference point (RP point) was established at the centroid of the end section. Constraints and loads were applied to the RP point under the following conditions: the bottom of the column was fully constrained in all degrees of freedom, while the top only released the in-plane rotational degrees of freedom and vertical translational degrees of freedom. A continuous vertical force was applied at the top of the column. The connection surface between the weld and the member was bounded by the binding constraint. A bilinear strengthening model was employed for the material constitutive model, considering the true stress and strain. The material properties obtained by the material property experiment were as follows: the yield stress (*f*_y_) was 450 MPa, the ultimate stress (*f*_u_) was 650 MPa, the elastic modulus (*E*) was 2.08 × 10^5^ MPa, the tangential modulus (*E*′) was 0.005E, and Poisson’s ratio (*ν*) was 0.3.

In this study, 60 models of eccentric RHS beam-column joints and 50 models of eccentric RHS beam–column joints with stiffening ribs were created to investigate the static performance of these joints. The models were developed by varying the geometric parameters such as the beam-column flange width ratio *β* (*b*/*B*), beam height to column flange width ratio *η* (*h*/*B*), column tube wall width-thickness ratio *γ* (*B*/2*T*), and beam-column section wall-thickness ratio *τ* (*t*/*T*). The objective was to examine the influence of these geometric parameters on the static performance of eccentric RHS beam-column joints.

Since the column members mostly had square cross-sections with equal heights and widths, this study focused on the case where the column width (*B*) was equal to the column height (*H*). The numerical models of the eccentric RHS beam-column joints were named following a specific format: for example, for “*J*-*P*-*p*1-*p*2”, “*J*” represents the joint type, “*P*” denotes the main geometric parameters studied in the group of models, “*p*1” denotes the column section width B in the group of models, and “*p*2” denotes specific values of the main variation parameters of the specimens. “+” represents the joint with stiffeners. Detailed information about the modeling, including the specifications of all numerical models of the eccentric RHS beam–column joints, can be found in Table 2 and Table 3.

## 3. Initial Stiffness of the Joint

### 3.1. Eccentric RHS Joints without Stiffeners

The calculation formula for the initial stiffness *K*_0_ of the T-shaped square tube intersecting joints was provided from the literature [2], as shown in Formula (1). It considers the deformation coefficient of the main panel *k*_cf_, the tensile deformation coefficient of the web *k*_cw_, and the shear deformation coefficient of the web *k*_sh_. The tensile deformation coefficient of the web can be calculated using Formulas (2) to (4).

(2) $$K_{0}=\frac{Eh^{2}}{\frac{2}{k_{\mathrm{cf}}}+\frac{2}{k_{\mathrm{cw}}}+\frac{1}{k_{\mathrm{sh}}}}$$

(3) $$k_{\mathrm{cw}}=\frac{2\cdot T\cdot b_{\mathrm{eff},\mathrm{cw},\mathrm{el}}}{H-3T}$$

(4) $$b_{\mathrm{eff},\mathrm{cw},\mathrm{el}}=2\cdot 0.7\cdot l_{\mathrm{eff},\mathrm{cw}}+t$$

(5) $$l_{\mathrm{eff},\mathrm{cw}}=\max\{T\cdot \sqrt{\frac{B}{2\cdot T}}\leq 2.5\cdot T,\frac{B}{2}\cdot \sqrt{1-\beta }\leq \frac{H}{2}\}$$

**Figure 3:**
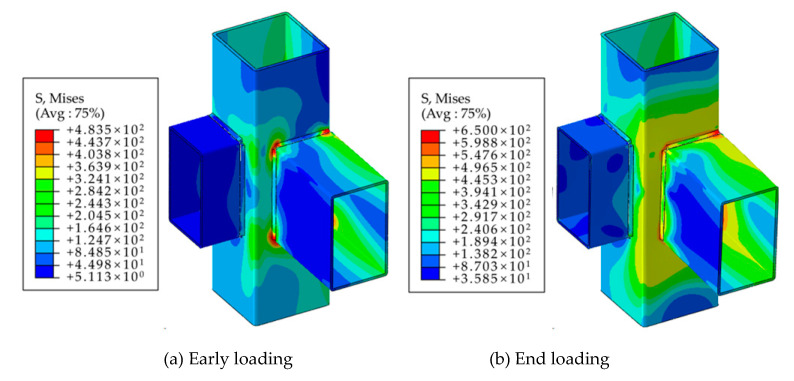
Stress distribution diagrams of joints without stiffeners.

The finite element calculation results are shown in Table 4, Table 5, Table 6 and Table 7. The stress distribution of the eccentric intersecting beam-column joint is depicted in Figure 3. Combining the test and finite element results [20] shows that the bending moment was primarily transmitted to the web on the eccentric side. The web’s local tensile and compressive deformations on the eccentric side contributed significantly to the joint’s overall deformation. Panel deformation mainly occurred towards the end of joint loading, particularly after the large area near the corner point where the web is connected to the beam, and entered the plastic range.

At the initial stage of bending, the mechanical behavior of eccentric RHS joints closely resembles that of equal-width T-joints with a flange width ratio (*β*) of 1. Taking *l*_eff_ = 2.5*T* and bringing it into Equation (4), because the wall thickness (*T*) is approximately equal to the wall thickness of the beam (*t*), and the column height (*H*) is much greater than (*t*), according to the forms of Formula (2)~Formula (5), the initial rotational stiffness *K*_0_ of the eccentric RHS joint can be written in the form of Formula (6). The coefficient (*k*) can be obtained through parameter analysis.

(6) $$K_{0}=kEhT^{2}$$

**Figure 4:**
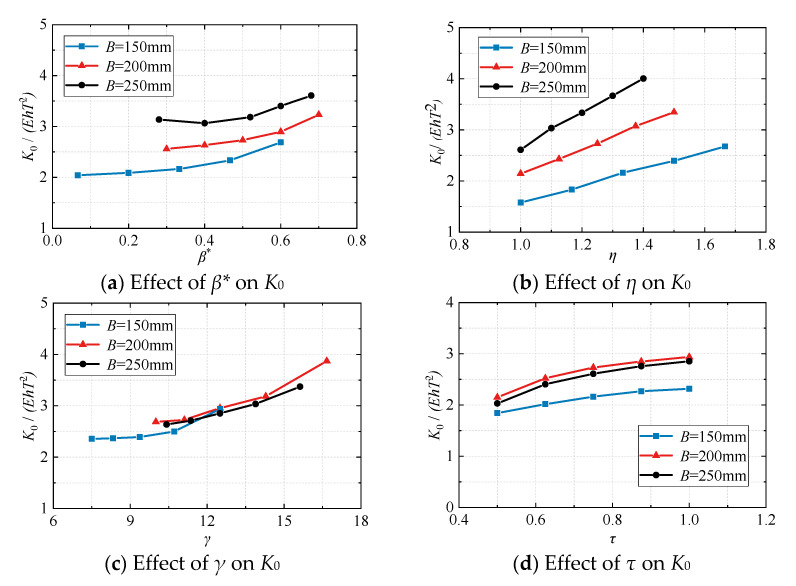
The effects of geometric parameters on the initial rotational stiffness *K*_0_.

The results of finite element parameter analysis are shown in Figure. 4. In Figure 4a, with an increase in the beam-to-column height-to-width ratio *η*(*h*/*B*), the bending moment was still borne by the eccentric side. However, the height of the beam increased, the stress area of the joint increased, and the development of plastic deformation became more complete. The initial rotational stiffness *K*_0_ of the joint increased linearly with an increase in *η*(*h*/*B*). The influence of *η*(*h*/*B*) can be fitted by a linear function. In Figure 4b, with an increase in the beam–column width ratio correction value β*(2*b*/*B* − 1), the width of the beam increased, and the bending moment was distributed more to the non-eccentric side. The initial rotational stiffness *K*_0_ of the joint increased non-linearly with an increase in β*(2*b*/*B* − 1). In Figure 4c,d, the failure mode of eccentric RHS joints was related to the thickness of the tube wall. Therefore, increasing the column diameter-thickness ratio *γ*(*B*/2*T*) is beneficial for enhancing the initial rotational stiffness *K*_0_. To some extent, increasing the beam-column wall thickness ratio *τ*(*t*/*T*) can also improve the initial rotational stiffness *K*_0_. The initial rotational stiffness *K*_0_ of the joint increased non−linearly with the *γ*(*B*/2*T*) and *τ*(*t*/*T*). The influence of β*(2*b*/*B* − 1), *γ*(*B*/2*T*), and *τ*(*t*/*T*) can be fitted by a quadratic function. The final coefficient (*k*) can be written in the form of Formula (7), where C_1_~C_8_ are undetermined constants. Using the data of 60 finite element models for parameter fitting, the fitting results are shown in Formula (8); most of the errors are less than 10%, and the maximum error is about 17%, which can obtain the initial rotational stiffness of the joint more accurately.

(7) $$k=(C_{1}+C_{2}\eta )\cdot (\beta^{*}{}^{2}+C_{3}\beta^{*}+C_{4})\cdot (\gamma^{2}+C_{5}\gamma +C_{6})\cdot (\tau^{2}+C_{7}\tau +C_{8})$$

(8) $$k=(-0.343+1.571\cdot \eta )\cdot (\beta^{*}{}^{2}-0.219\cdot \beta^{*}+1.211)\cdot (\gamma^{2}+7.620\cdot \gamma +236{.060C}_{6})\cdot (\tau^{2}+1080.537\cdot \tau +1710.577)\cdot 10^{-6}$$

### 3.2. Eccentric RHS Joints with Stiffeners

According to the component method, the presence of a stiffener in the joint can be considered as an equivalent spring connected in parallel at the joint. This additional spring enhances the rotational stiffness of the joint. By analyzing the stiffness contributed by the stiffener, we can determine the overall stiffness of the joint with the stiffener. This stiffness is then combined with the stiffness of the joints without a stiffener to obtain the total rotational stiffness of the joint. Detailed information about the modeling, including the specifications of all numerical models of eccentric RHS beam-column joints, can be found in Table 8.

**Figure 5:**
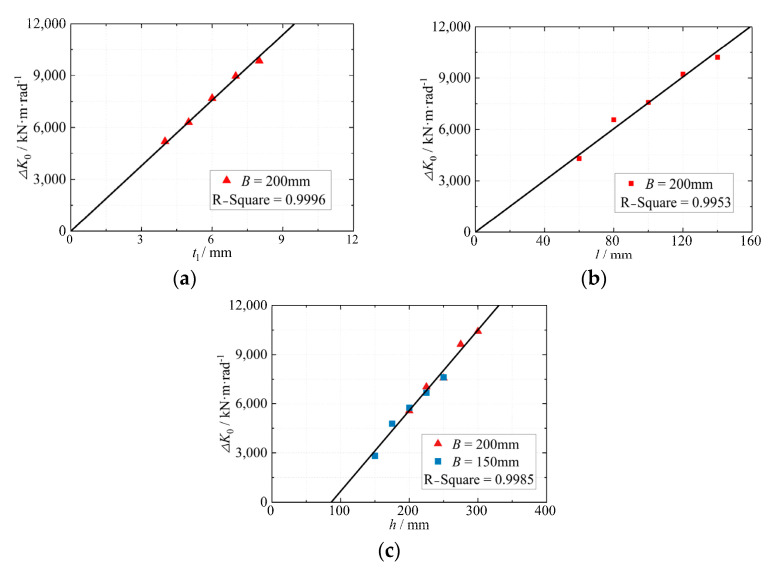
Effects of different stiffeners on joint rotational stiffness increments Δ*K*_0_; (**a**) Effect of *t*_1_ on Δ*K*_0_; (**b**) Effect of *l* on Δ*K*_0_; (**c**) Effect of *h* on Δ*K*_0_.

Formula (9) represents the incremental value of the initial rotational stiffness (Δ*K*_0_) resulting from the presence of a stiffener in the eccentric RHS joints. By analyzing the parameters that influence the increase in bearing capacity, the main factors affecting Δ*K*_0_ can be determined. The geometric parameters, namely the stiffener thickness (*t*_l_), beam height (*h*), and length (*l*), were investigated, as shown in Figure 5. It was observed that Δ*K*_0_ is directly proportional to *t*_l_ and *l*, indicating that larger dimensions of the gusset plate lead to a more pronounced enhancement in joint stiffness through the stiffener. Furthermore, Δ*K*_0_ exhibits a linear relationship with the beam height (*h*), with a positive intersection point on the x-axis. This signifies that the stiffener’s ability to improve the joint stiffness may be limited when the beam height is small. This can be attributed to the reduced longitudinal deformation along the beam direction, which hinders the effective contribution of the stiffeners.

(9) $$\Delta K_{0}=\frac{\Delta M}{\theta }$$

**Figure 6:**
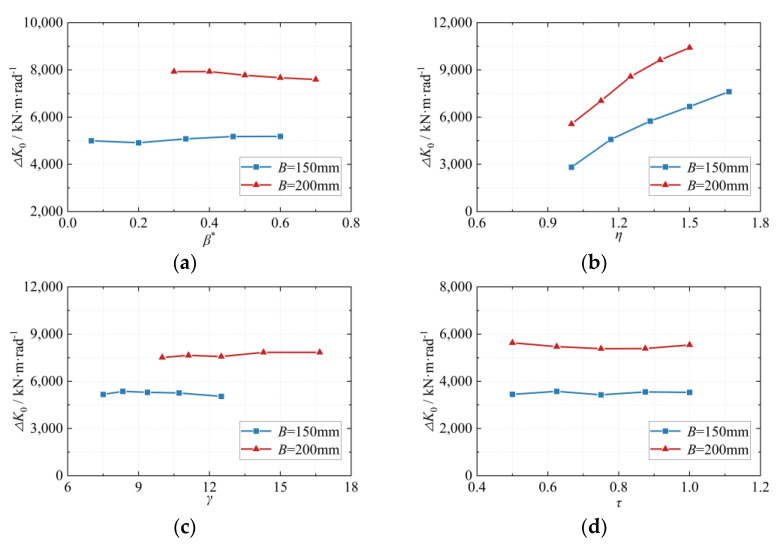
The effects of geometric parameters on the rotational stiffness increment Δ*K*_0_. (**a**) Effect of β* on Δ*K*_0_; (**b**) Effect of *η* on Δ*K*_0_; (**c**) Effect of *γ* on Δ*K*_0_; (**d**) Effect of *τ* on Δ*K*_0_.

The parametric analysis results of the geometric parameters influencing the joint dimensions are presented in Figure 6. Variations in the beam height or the beam-to-column flange width ratio *η* were observed to have a more significant impact on the increment of the initial rotational stiffness (Δ*K*_0_). Conversely, changes in other geometric parameters of the beam-column members and modifications to the stiffener shape exhibited a relatively smaller effect on Δ*K*_0_. Notably, the parameter η was primarily achieved by altering the beam height (*h*), which was not explicitly considered in the final formula to avoid redundancy.

Finally, the increment of the initial rotational stiffness (Δ*K*_0_) of the joint is expressed by Formula (10), and a parameter fitting was performed using the data from 50 finite element models established in this research. The fitting result is presented in Formula (10). When calculating the effect of the stiffener on the rotational stiffness, the contribution of the stiffener was ignored if the calculated Δ*K*_0_ was negative. The effect of the stiffener on the joint rotational stiffness was calculated using Formula (11), while the initial rotational stiffness of the joint without a stiffener was calculated using Formula (8). These two values were then added together to obtain the initial rotational stiffness of the joint with a stiffener. The maximum error (Δ_max_) was −11.21%, and the average error (Δ_avg_) was −0.24%, demonstrating that the results met the engineering accuracy requirements.

(10) $$\Delta K_{0}=C_{1}Et_{l}l(h+C_{2})$$

(11) $$\Delta K_{0}=0.440\cdot Et_{l}l\cdot (h-105.57)$$

## 4. Spatial Effect

Compared with in-plane joints, the bearing capacity of spatial joints is influenced by two additional factors: geometric effect and load effect. These two influencing factors are usually collectively referred to as the spatial effect. This section investigates the spatial effects of joints under complex force conditions through building finite element models.

### 4.1. Geometric Effect

**Figure 7:**
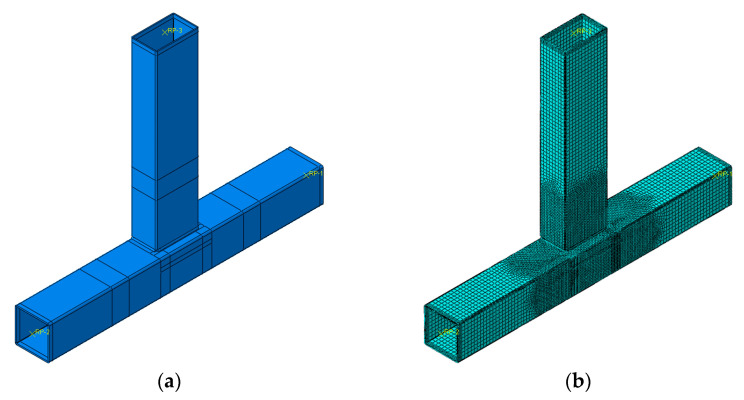
Finite element models (geometric effect). (**a**) Geometric model; (**b**) Mesh of the model.

The geometric effect pertains to the influence of the branch tubes’ presence on the stiffness of the joints and, consequently, on the ultimate bearing capacity of the joints. In order to figure out the geometric effect on the eccentric RHS beam-column joints, finite element models as shown in Figure 7 were established.

**Figure 8:**
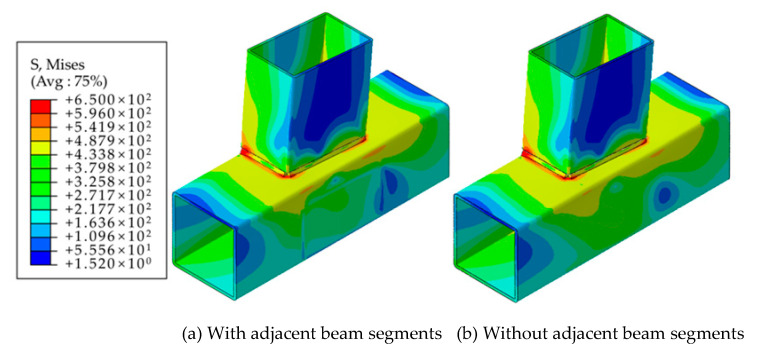
Comparison of finite element results (geometric effect).

Figure 8 presents the comparison results of the impact of adjacent beam segments on the core region of the joint area. It was observed that under the same applied load, the stress distribution in the two models was nearly identical. This indicates that the presence of adjacent beam segments does not significantly affect the stress distribution within the joint area. The finite element results revealed that during in-plane bending of the eccentric RHS beam-column joints, the primary force was mainly borne by the two column plates directly connected to the beam. The stress level in the plate connected to the adjacent beam section was relatively lower. Consequently, the presence of adjacent beam sections did not provide effective constraints on the main stressed tube wall or contribute to the improvement in joint stiffness.

**Figure 9:**
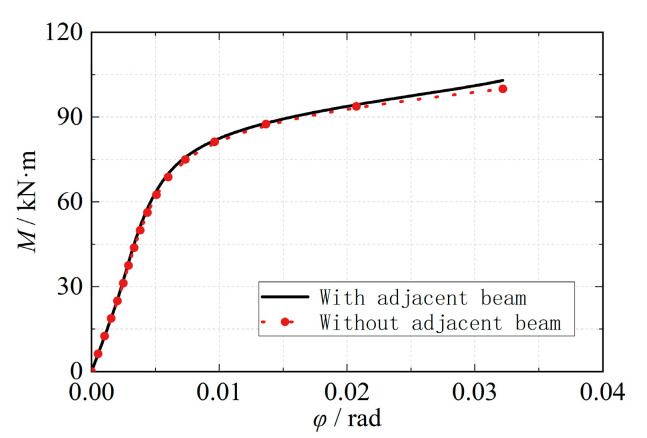
Bending moment-angle curves (geometric effect).

Figure 9 illustrates the comparison results of the moment-rotation angle curves for the two cases. It can be observed that the adjacent beam segments only exerted a certain restraining effect on the core region of the joint area when the joint reached its ultimate bearing capacity and undergoes significant deformation; however, this effect was not significant. Consequently, the influence of the geometric effect on the bearing performance of the eccentric RHS beam-column joint was considered negligible.

### 4.2. Load Effect

**Figure 10:**
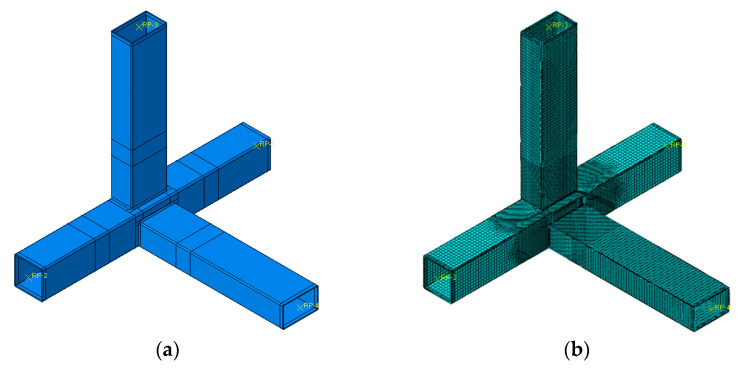
Finite element models (load effect). (**a**) Geometric model; (**b**) Mesh of the model.

The load effect refers to the influence of changes in the load direction and magnitude applied to the branch tube on the ultimate bearing capacity of the joint. In order to figure out the load effects of the joints on the eccentric RHS beam-column joints, finite element models as shown in Figure 10 were established.

**Figure 11:**
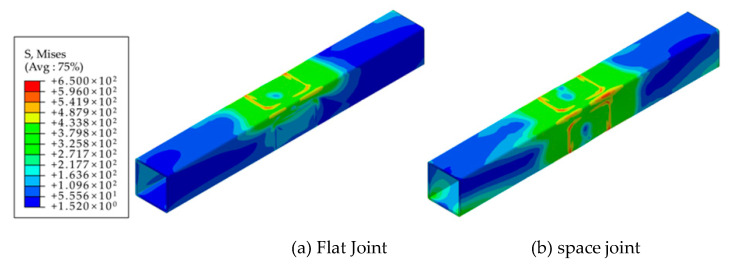
Comparison of finite element results (load effect).

Figure 11a shows the finite element analysis results that depict the eccentric RHS beam-column joints as plane joints subjected to in-plane bending moments. Under this loading condition, it was observed that the two column plates directly connected to the beam bore the majority of the load, while the stress levels in the other two plates were relatively lower. Figure 11b presents the finite element analysis results when the joint was subjected to both in-plane and out-of-plane bending moments. In this case, where the joint was loaded simultaneously by two beams, it was observed that the stress distribution and plastic deformation development in the two plates corresponding to one side beam were similar to those in the case of a single beam. This indicates that when multiple beams are loaded simultaneously, the influence between the members is relatively small, and the load effect on the joint as a spatial joint is also limited.

**Figure 12:**
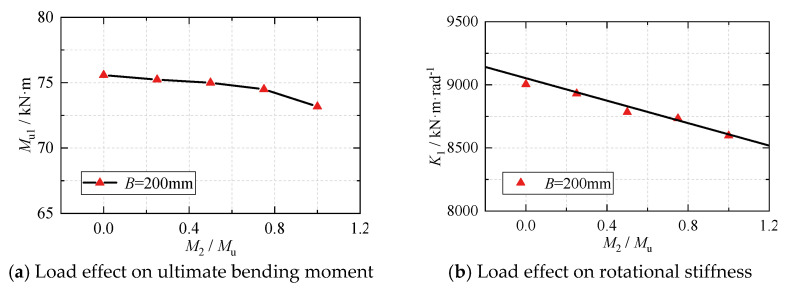
The influence of load effect on the bearing capacity of joints.

By varying the bending moment *M*_2_ of the adjacent side beam, the bending capacity *M*_u1_ and initial rotational stiffness *K*_1_ of the joint in the main stress direction were analyzed. The bending capacity *M*_u_ represents the joint’s ultimate bearing capacity when only a single beam of the joint is subjected to load. The results of this analysis are shown in Figure 12. 

The ultimate bending moment of the joint on the other side was observed to be minimally affected when the adjacent side beam’s bending moment was low. However, a significant decrease in joint bearing capacity was observed when the bending moment of the adjacent side beam exceeded 80% of the ultimate bending moment. When the bending moment of the adjacent side beam was equal to the ultimate bearing capacity of the single beam joint, the joint’s bearing capacity and stiffness experienced a degradation of approximately 5%. However, in practical applications, it is unlikely that the beams on both sides of the joint structure will be loaded close to their ultimate bearing capacities simultaneously. Therefore, the load effect on the bearing capacity of the eccentric RHS beam-column joint can be disregarded in the design process, as the beams on both sides of the joint structure are not expected to simultaneously be subjected to loads approaching their ultimate bearing capacities.

## 5. Mathematical Model of Eccentric RHS Beam-Column Joint

### 5.1. Selection of Mathematical Model

**Figure 13:**
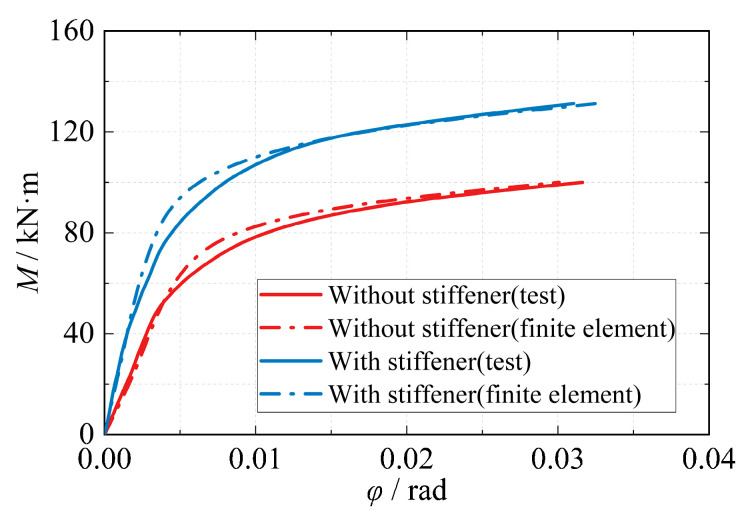
Test and finite element moment-rotation curves.

According to the literature [20] and the analysis in this research, it is known that the final damage mode of the joint is large deformation damage when the eccentric RHS beam-column joint is subjected to in-plane unidirectional moment loading. The moment-rotation relationship curve is composed of three parts: an initial elastic section, a non-linear transition section, and a strengthening section which are showed in Figure 13. The relationship between moment and rotation is approximately linear in the initial elastic range. As the joint gradually transitions to the strengthening range, the slope of the curve decreases. After the joint reaches its ultimate bearing capacity, there is no significant change in the moment with increasing rotation within a large deformation range.

**Figure 14:**
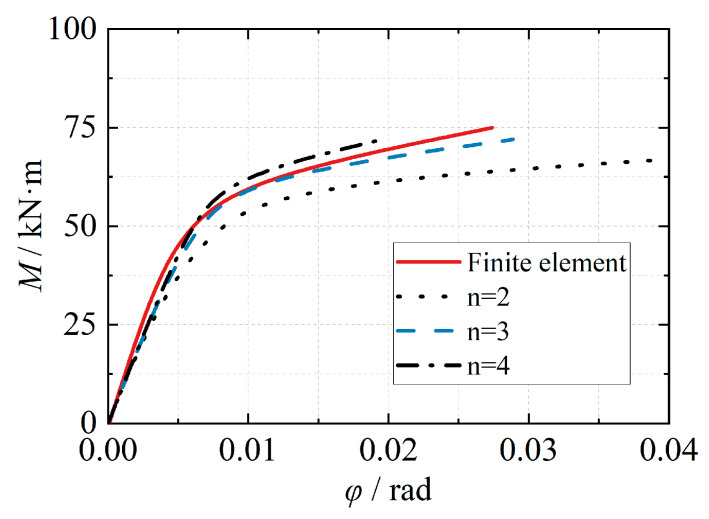
Effect of different shape coefficients *n*.

**Figure 15:**
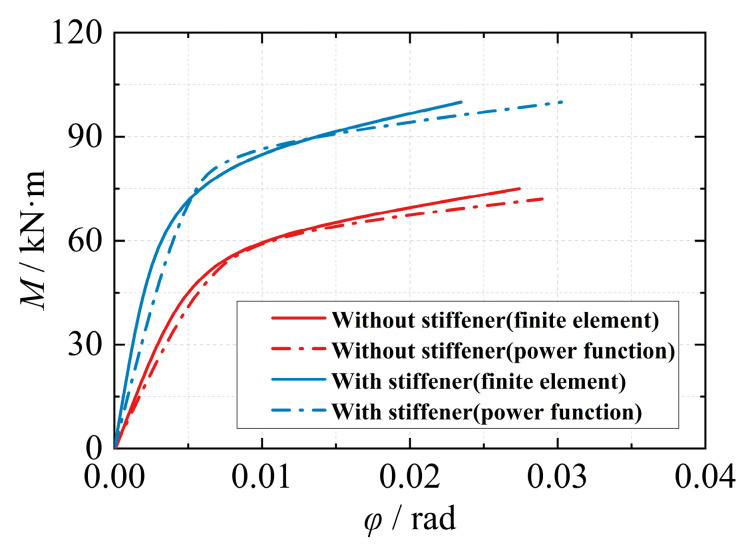
Comparison of model results and finite element results.

The power function model curve exhibits a smaller slope variation at lower rotations. As the rotation increases, the slope decreases gradually. The moment gradually converges to the ultimate flexural capacity, and the trend of the power function model is consistent with the variation observed in the moment-rotation curve obtained from experiments and finite element analysis. Therefore, the power function model (Formula (9)) can effectively simulate the entire loading process of the joint. Figure 14 presents the predicted results of the power function model with different shape coefficients (*n*). When the shape coefficient of the power function model is set to 3, it provides a good simulation of the joint’s loading process. When applying this model to the strengthened ribbed joint of the eccentric RHS joint, the moment-rotation curve of the joint closely matches the calculated results obtained from finite element analysis (Figure 15).

(12) $$\theta_{r}=\frac{M}{R_{\mathrm{ki}}}\cdot \frac{1}{{\left[1-{\left(M/M_{u}\right)}^{n}\right]}^{1/n}}$$

(13) $$\theta =\frac{M}{K_{0}}\cdot \frac{1}{{\left[1-{\left(M/M_{u}\right)}^{3}\right]}^{1/3}}$$

### 5.2. Joint-Level Finite Element Verification

Directly using solid elements for modeling requires significant computational time when conducting structural calculations. It is common in the design process to employ a beam system model for the overall analysis and design of the structure. The effect of different joint connection types on the final calculation results in frame structures cannot be simply ignored. Therefore, it is necessary to address how to incorporate the effect of joint semi-rigidity into the beam system model.

The mathematical model of the eccentric RHS joint’s static performance under in-plane bending was established in this study. A rotational spring element was introduced at the intersection of the beam and column components to account for the effect of joint semi-rigidity in the beam system model. This method uses beam system units instead of solid units for finite element modeling, which greatly reduces the computational complexity and improves the computational efficiency.

**Figure 16:**
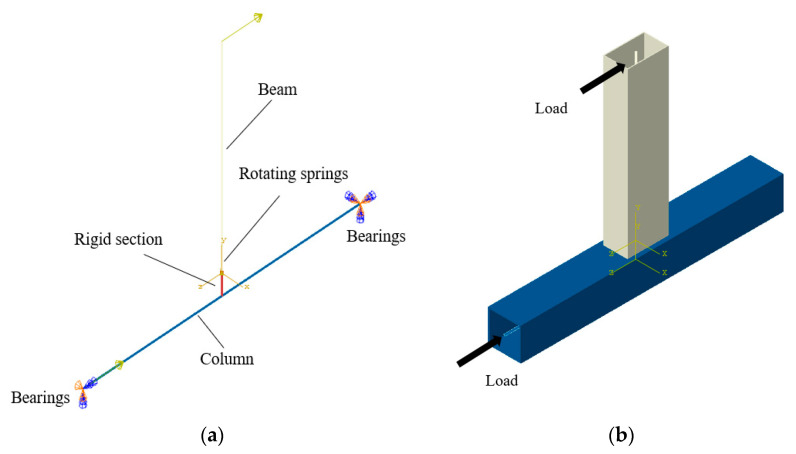
Finite element model of bar system. (**a**) Thumbnail; (**b**) Section rendering.

**Figure 17:**
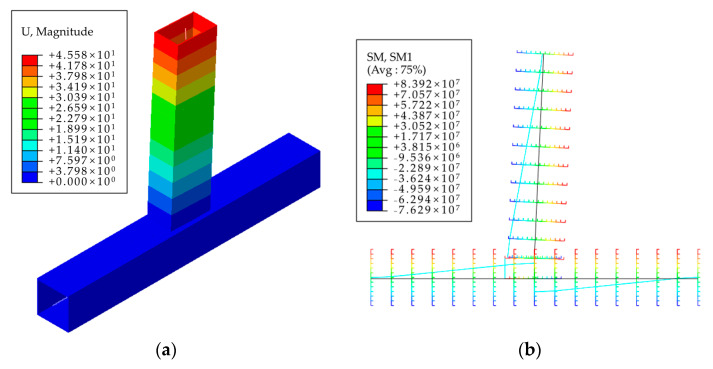
Calculation results of the beam system finite element model. (**a**) deformation diagram; (**b**) bending moment diagram.

The beam system model established in this study is shown in Figure 16a. The column component is a rectangular hollow section (200 mm × 8 mm), and the beam component is a rectangular hollow section (250 mm × 150 mm × 6 mm). A rigid section connects the column and beam components with the same cross-sectional dimensions as the beam section. Non-linear rotational springs were set up at the intersection of beam-column members to simulate semi-rigid joints, which uses ABAQUS’ own Connector module. The finite element model of the beam system is shown in Figure 17.

**Figure 18:**
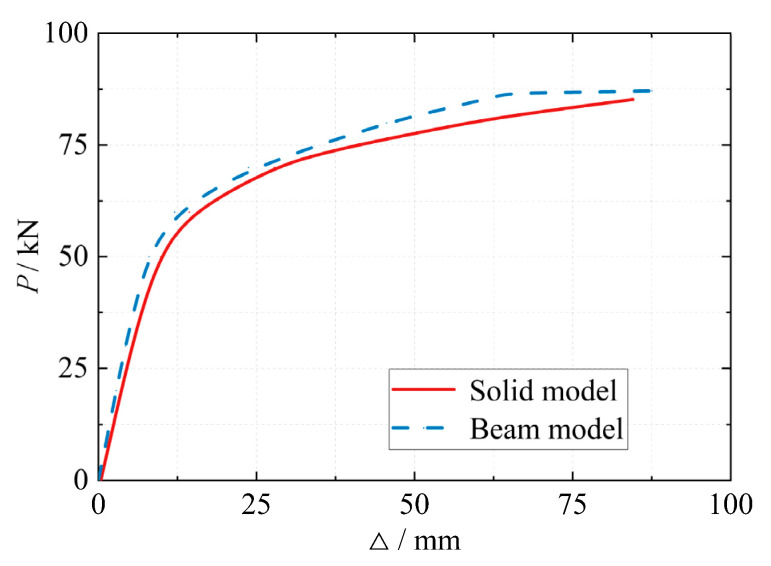
Load-displacement curves of beam system model and solid model.

The calculation results of the finite element model of the beam system are shown in Figure 17. The ultimate bearing capacity of the joint is 76.29 kN·m, which is only 1% error compared with the bearing capacity calculation result of the solid model 75.58 kN·m. Figure 18 shows a comparison of the calculation results of the load-displacement curve between the beam system model and the solid model. In the figure, *P* is the vertical load at the loading point at the beam end, and Δ is the vertical displacement at the loading point at the beam end. It can be seen from Figure 18 that the calculation results of the beam system model and the solid model are basically consistent after using the power function model to simulate the semi-rigidity of the joints. Throughout the whole process, the model’s accuracy was effectively guaranteed, but the calculation time of the beam system model was only 1% of the solid model; the computational efficiency was significantly improved.

In the case of the solid models, when the load on the joint reached the ultimate carrying capacity, the load still increased slightly with displacement. However, when the load on the joint reached the ultimate carrying capacity, the load could not be significantly increased in the case of the beam system models. This limitation arose from using the power function model, where the maximum load-bearing capacity that the joint could achieve was the ultimate carrying capacity. The model did not account for any strengthening effect that may have occurred after the joint reached its ultimate carrying capacity.

### 5.3. Structure-Level Finite Element Verification

**Figure 19:**
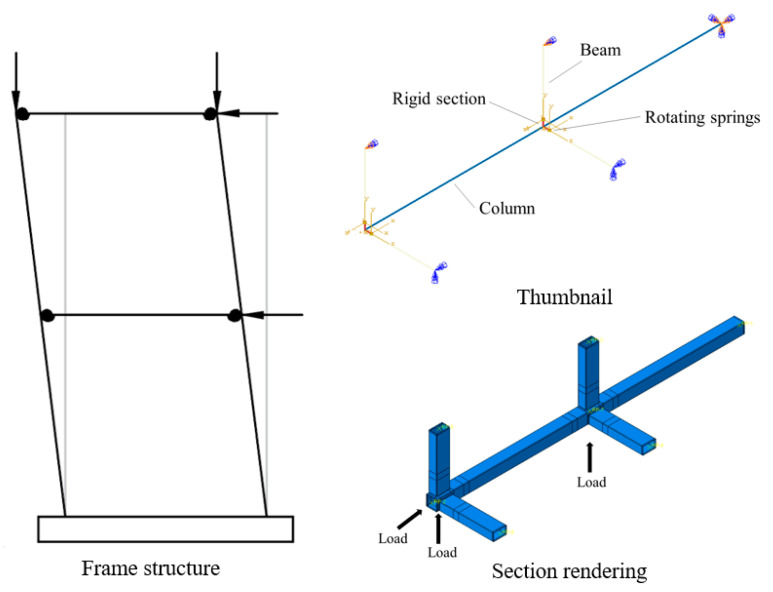
Finite element model of frame structure.

To validate the mathematical model’s accuracy for the joint’s static performance at the structural level, a finite element model of a frame structure was established. The 1/4th structure was considered to simplify the calculations, as shown in Figure 19. The modeling approach used in Section 5.2 was employed at the connections of the frame beams and columns, where the joint semi-rigidity was simulated using non-linear rotational springs. A vertical load was applied to the column components of the frame structure to simulate the effects of permanent and live loads. Additionally, an equal horizontal load was applied at each joint of the structure to simulate the effects of wind loads until structural failure occurred.

**Figure 20:**
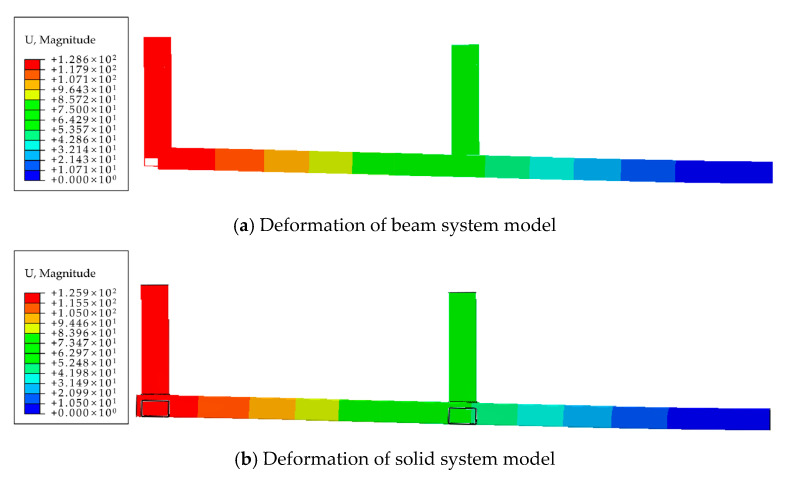
Deformation diagrams of frame structure finite element model.

**Figure 21:**
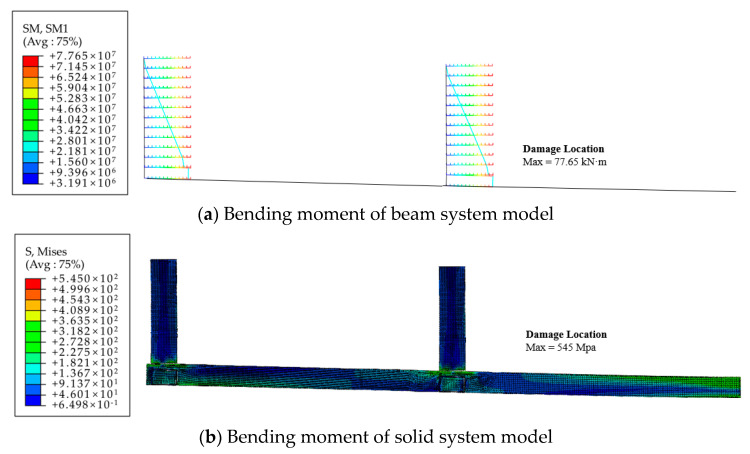
Damage positions of frame structure finite element model.

The finite element analysis results of the frame structure are shown in Figure 20 and Figure 21. In both models, the locations of structural failure occurred at the joints of the first-layer beams and columns. At the point of failure, the moments at both joints exceeded the ultimate carrying capacity, reaching 77.65 kN·m, with the maximum stress reaching 545 MPa.

The beam model yielded the following results for the maximum displacements: 75 mm for the first layer, and 129 mm for the second layer. On the other hand, the solid model produced the following results: 73 mm for the first layer, and 126 mm for the second layer. The calculated results from both modeling approaches are essentially identical, with a small discrepancy in the displacement calculations of only 2.67% and 2.34% for the beam and solid models, respectively. This demonstrates the effectiveness of the proposed simplified calculation method for the eccentric RHS joint when applied to frame structure analysis.

## 6. Conclusions

This research comprehensively investigated the in-plane bearing capacity of eccentric RHS beam-column joints. We analyzed the formula for the initial rotational stiffness of the joint, and explored the influence of spatial effects on its performance. The following conclusions can be drawn:

(1) The rotational stiffness (*K*_0_) of eccentric RHS joints is primarily influenced by the tension-compression deformation stiffness (*k*_cw_) of the web. It increases with the height-to-column flange width ratio (*η*) and the beam-to-column wall thickness ratio (*τ*). Meanwhile, the column’s wall width-to-thickness ratio (*γ*) increases with these ratios’ increments.
(2) The rotational stiffness (*K*_0_) does not change significantly when the beam-column flange width ratio (*β*) is less than 0.5. It increases significantly with an increase in (*β*) when (*β*) is greater than 0.5, indicating that the bending moment distribution form of the joint changes.
(3) Considering the force mechanism of the eccentric RHS joint, the side plate connected to the stressed beam and the web plate on the eccentric side bear most of the load, while the plates on the other two sides of the rectangular tubular column member are minimally affected. Therefore, for a T-shaped space joint with a 90° included angle, the mutual influence between the two stressed beams can be disregarded.
(4) To simulate the semi-rigid effect of the joint, this study adopted a nonlinear corner spring model. Compared with the solid element analysis, the ultimate bending moment error of the joint is only 1.0%, the average lateral displacement error in the frame structure is only 2.5%. The finite element analysis confirmed the accuracy of the power function model in accurately simulating the static load behavior of the joint, particularly the bending moment-angle relationship.

## Figures and Tables

**Table 1:** The ultimate bending moments obtained from finite element analysis.

Maximum Mesh Size	Ultimate Bending Moment *M*_u_/(kN/m)	Error/%
80	96.18	0.81
90	96.43	1.07
100	98.12	2.84
110	101.53	6.41
120	106.23	11.34

**Table 2:** Eccentric RHS joints without stiffeners.

Model Number	Column Size		Beam Size		
	Width B/mm	Thickness T/mm	Width b/mm	Height h/mm	Thickness t/mm
J-*β*-200-130	200	8	130	250	6
J-*β*-200-140	200	8	140	250	6
J-*β*-200-150	200	8	150	250	6
J-*β*-200-160	200	8	160	250	6
J-*β*-200-170	200	8	170	250	6
J-*β*-150-80	150	8	80	200	6
J-*β*-150-90	150	8	90	200	6
J-*β*-150-100	150	8	100	200	6
J-*β*-150-110	150	8	110	200	6
J-*β*-150-120	150	8	120	200	6
J-*β*-250-160	250	8	160	300	6
J-*β*-250-175	250	8	175	300	6
J-*β*-250-190	250	8	190	300	6
J-*β*-250-200	250	8	200	300	6
J-*β*-250-210	250	8	210	300	6
J-*γ*-200-6	200	6	150	250	6
J-*γ*-200-7	200	7	150	250	7
J-*γ*-200-8	200	8	150	250	8
J-*γ*-200-9	200	9	150	250	9
J-*γ*-200-10	200	10	150	250	10
J-*γ*-150-6	150	6	100	200	6
J-*γ*-150-7	150	7	100	200	7
J-*γ*-150-8	150	8	100	200	8
J-*γ*-150-9	150	9	100	200	9
J-*γ*-150-10	150	10	100	200	10
J-*γ*-250-8	250	8	180	300	8
J-*γ*-250-9	250	9	180	300	9
J-*γ*-250-10	250	10	180	300	10
J-*γ*-250-11	250	11	180	300	11
J-γ-250-12	250	12	180	300	12
J-η-200-200	200	8	150	200	6
J-*η*-200-225	200	8	150	225	6
J-*η*-200-250	200	8	150	250	6
J-*η*-200-275	200	8	150	275	6
J-*η*-200-300	200	8	150	300	6
J-*η*-150-150	150	8	100	150	6
J-*η*-150-175	150	8	100	175	6
J-*η*-150-200	150	8	100	200	6
J-*η*-150-225	150	8	100	225	6
J-*η*-150-250	150	8	100	250	6
J-*η*-250-250	250	8	200	250	6
J-*η*-250-275	250	8	200	275	6
J-*η*-250-300	250	8	200	300	6
J-*η*-250-325	250	8	200	325	6
J-*η*-250-350	250	8	200	350	6
J-*τ*-200-4	200	8	150	250	4
J-*τ*-200-5	200	8	150	250	5
J-*τ*-200-6	200	8	150	250	6
J-*τ*-200-7	200	8	150	250	7
J-*τ*-200-8	200	8	150	250	8
J-*τ*-150-4	150	8	100	200	4
J-*τ*-150-5	150	8	100	200	5
J-*τ*-150-6	150	8	100	200	6
J-*τ*-150-7	150	8	100	200	7
J-*τ*-150-8	150	8	100	200	8
J-*τ*-250-4	250	8	200	250	4
J-*τ*-250-5	250	8	200	250	5
J-*τ*-250-6	250	8	200	250	6
J-*τ*-250-7	250	8	200	250	7
J-*τ*-250-8	250	8	200	250	8

**Table 3:** Eccentric RHS joints with stiffeners.

Model Number	Column Size		Beam Size			Stiffener Size	
	Width B/mm	Thickness T/mm	Width b/mm	Height h/mm	Thickness t/mm	Thickness *t*_l_/mm	Length *l*/mm
J-*t*_1_-200-4+	200	8	150	250	8	4	100
J-*t*_1_-200-5+	200	8	150	250	8	5	100
J-*t*_1_-200-6+	200	8	150	250	8	6	100
J-*t*_1_-200-7+	200	8	150	250	8	7	100
J-*t*_1_-200-8+	200	8	150	250	8	8	100
J-*l*-200-60+	200	8	150	250	6	6	60
J-*l*-200-80+	200	8	150	250	6	6	80
J-*l*-200-100+	200	8	150	250	6	6	100
J-*l*-200-120+	200	8	150	250	6	6	120
J-*l*-200-140+	200	8	150	250	6	6	140
J-*β*-200-130+	200	8	130	250	6	6	100
J-*β*-200-140+	200	8	140	250	6	6	100
J-*β*-200-150+	200	8	150	250	6	6	100
J-*β*-200-160+	200	8	160	250	6	6	100
J-*β*-200-170+	200	8	170	250	6	6	100
J-*β*-150-80+	150	8	80	200	6	6	100
J-*β*-150-90+	150	8	90	200	6	6	100
J-*β*-150-100+	150	8	100	200	6	6	100
J-*β*-150-110+	150	8	110	200	6	6	100
J-*β*-150-120+	150	8	120	200	6	6	100
J-*η*-200-200+	200	8	150	200	6	6	100
J-*η*-200-225+	200	8	150	225	6	6	100
J-*η*-200-250+	200	8	150	250	6	6	100
J-*η*-200-275+	200	8	150	275	6	6	100
J-*η*-200-300+	200	8	150	300	6	6	100
J-*η*-150-150+	150	8	100	150	6	6	100
J-*η*-150-175+	150	8	100	175	6	6	100
J-*η*-150-200+	150	8	100	200	6	6	100
J-*η*-150-225+	150	8	100	225	6	6	100
J-*η*-150-250+	150	8	100	250	6	6	100
J-*γ*-200-6+	200	6	150	250	6	6	100
J-*γ*-200-7+	200	7	150	250	6	6	100
J-*γ*-200-8+	200	8	150	250	6	6	100
J-*γ*-200-9+	200	9	150	250	6	6	100
J-*γ*-200-10+	200	10	150	250	6	6	100
J-*γ*-150-6+	150	6	100	200	6	6	100
J-*γ*-150-7+	150	7	100	200	6	6	100
J-*γ*-150-8+	150	8	100	200	6	6	100
J-*γ*-150-9+	150	9	100	200	6	6	100
J-*γ*-150-10+	150	10	100	200	6	6	100
J-*γ*-200-6+	200	6	150	250	6	6	100
J-*τ*-200-4+	200	8	150	250	4	4	100
J-*τ*-200-5+	200	8	150	250	5	4	100
J-*τ*-200-6+	200	8	150	250	6	4	100
J-*τ*-200-7+	200	8	150	250	7	4	100
J-*τ*-200-8+	200	8	150	250	8	4	100
J-*τ*-150-4+	150	8	100	200	4	4	100
J-*τ*-150-5+	150	8	100	200	5	4	100
J-*τ*-150-6+	150	8	100	200	6	4	100
J-*τ*-150-7+	150	8	100	200	7	4	100
J-*τ*-150-8+	150	8	100	200	8	4	100

**Table 4:** Stiffnesses at different beam-column flange width ratios.

Model Number	Beam–Column Flange Width Ratio *β*	Rotational Stiffness *K*_0_/(kN·m·rad^−1^)		
		Finite Element	Formula	Error/%
J-*β*-200-130	0.65	8435.53	8108.01	−3.88
J-*β*-200-140	0.70	8681.78	8423.72	−2.97
J-*β*-200-150	0.75	9004.52	8870.70	−1.49
J-*β*-200-160	0.80	9549.81	9448.95	−1.06
J-*β*-200-170	0.85	10,651.09	10,158.47	−4.63
J-*β*-150-80	0.53	5386.18	5526.39	2.60
J-*β*-150-90	0.60	5508.9	5555.64	0.85
J-*β*-150-100	0.67	5710.09	5748.51	0.67
J-*β*-150-110	0.73	6156.72	6105.02	−0.84
J-*β*-150-120	0.80	7090.71	6625.16	−6.57
J-*β*-250-160	0.64	12,403.06	11,312.82	−8.79
J-*β*-250-175	0.70	12,123.24	11,822.41	−2.48
J-*β*-250-190	0.76	12,589.15	12,597.31	0.06
J-*β*-250-200	0.80	13,458.62	13,261.29	−1.47
J-*β*-250-210	0.84	14,268.36	14,043.19	−1.58

**Table 5:** Stiffnesses at different beam height to column flange width ratios.

Model Number	Beam Height to Column Flange Width Ratio *η*	Rotational Stiffness *K*_0_/(kN·m·rad^−1^)		
		Finite Element	Formula	Error/%
J-*η*-200-200	1.00	5655.36	5376.64	−4.93
J-*η*-200-225	1.13	6909.83	7016.18	1.54
J-*η*-200-250	1.25	9004.52	8870.70	−1.49
J-*η*-200-275	1.38	10,786.44	10,940.21	1.43
J-*η*-200-300	1.50	13,237.56	13,224.70	−0.10
J-*η*-150-150	1.00	3121.51	3022.32	−3.18
J-*η*-150-175	1.17	4228.32	4278.00	1.17
J-*η*-150-200	1.33	5703.28	5748.51	0.79
J-*η*-150-225	1.50	7100.36	7433.88	4.70
J-*η*-150-250	1.67	8818.22	9334.08	5.85
J-*η*-250-250	1.00	8602.05	8799.27	2.29
J-*η*-250-275	1.10	11,142.91	10,917.69	−2.02
J-*η*-250-300	1.20	13,189.28	13,261.29	0.55
J-*η*-250-325	1.30	15,711.35	15,830.08	0.76
J-*η*-250-350	1.40	18,475.05	18,624.04	0.81

**Table 6:** Stiffnesses at different column tube wall width–thickness ratios.

Model Number	Column Tube Wall Width–Thickness Ratio *γ*	Rotational Stiffness *K*_0_/(kN·m·rad^−1^)		
		Finite Element	Formula	Error/%
J-*γ*-200-6	16.67	7164.19	7262.48	1.37
J-*γ*-200-7	14.29	7785.08	7999.25	2.75
J-*γ*-200-8	12.50	9004.52	8870.70	−1.49
J-*γ*-200-9	11.11	9973.64	9862.26	−1.12
J-*γ*-200-10	10.00	11,492.77	10,965.21	−4.59
J-*γ*-150-6	12.50	4347.77	4415.37	1.55
J-*γ*-150-7	10.71	4694.31	5035.76	7.27
J-*γ*-150-8	9.38	5710.09	5748.51	0.67
J-*γ*-150-9	8.33	6901.66	6547.09	−5.14
J-*γ*-150-10	7.50	8195.46	7427.57	−9.37
J-*γ*-250-8	15.63	12,335.87	12,051.23	−2.31
J-*γ*-250-9	13.89	14,203.56	13,124.57	−7.60
J-*γ*-250-10	12.50	14,641.01	14,333.68	−2.10
J-*γ*-250-11	11.36	16,173.1	15,668.97	−3.12
J-*γ*-250-12	10.42	17,516.66	17,124.04	−2.24

**Table 7:** Stiffnesses at different beam–column section wall–thickness ratios.

Model Number	Beam–Column Section Wall–Thickness Ratio *τ*	Rotational Stiffness *K*_0_/(kN·m·rad^−1^)		
		Finite Element	Formula	Error/%
J-*τ*-200-4	0.50	7085.27	7919.16	11.77
J-*τ*-200-5	0.63	8310.69	8394.87	1.01
J-*τ*-200-6	0.75	9004.52	8870.70	−1.49
J-*τ*-200-7	0.88	9389.58	9346.63	−0.46
J-*τ*-200-8	1.00	9683.7	9822.67	1.44
J-*τ*-150-4	0.50	4863.35	5131.88	5.52
J-*τ*-150-5	0.63	5318.5	5440.16	2.29
J-*τ*-150-6	0.75	5703.28	5748.51	0.79
J-*τ*-150-7	0.88	5983.3	6056.94	1.23
J-*τ*-150-8	1.00	6110.39	6365.43	4.17
J-*τ*-250-4	0.50	6695.22	7855.39	17.33
J-*τ*-250-5	0.63	8124.95	8327.28	2.49
J-*τ*-250-6	0.75	8602.05	8799.27	2.29
J-*τ*-250-7	0.88	9223.87	9271.37	0.51
J-*τ*-250-8	1.00	9604.13	9743.58	1.45

**Table 8:** Finite element calculation results of eccentric RHS joints with stiffeners.

Model Number	Rotational Stiffness *K*_0_/(kN·m·rad^−1^)		
	Finite Element	Formula	Error/%
J-*t*_1_-200-4+	14,880.21	14,915.25	0.24
J-*t*_1_-200-5+	15,987.88	16,223.14	1.47
J-*t*_1_-200-6+	17,373.82	17,531.02	0.90
J-*t*_1_-200-7+	18,650.79	18,838.91	1.01
J-*t*_1_-200-8+	19,533.17	20,146.8	3.14
J-*l*-200-60+	13,313.45	13,712.91	3.00
J-*l*-200-80+	15,567.45	15,282.38	−1.83
J-*l*-200-100+	16,579.87	16,851.84	1.64
J-*l*-200-120+	18,229.8	18,421.31	1.05
J-*l*-200-140+	19,216.52	19,990.77	4.03
J-*β*-200-130+	16,367.13	16,282.85	−0.51
J-*β*-200-140+	16,712.04	16,529.1	−1.09
J-*β*-200-150+	16,579.71	16,851.84	1.64
J-*β*-200-160+	17,218.84	17,397.13	1.04
J-*β*-200-170+	18,244.75	18,498.41	1.39
J-*β*-150-80+	9700.97	10,332.07	6.51
J-*β*-150-90+	10,422.72	10,639.5	2.08
J-*β*-150-100+	10,790.95	10,840.69	0.46
J-*β*-150-110+	11,720.02	11,271.38	−3.83
J-*β*-150-120+	12,227.62	12,173.72	−0.44
J-*η*-200-200+	11,217.62	10,785.96	−3.85
J-*η*-200-225+	13,943.22	13,398.79	−3.90
J-*η*-200-250+	16,579.71	16,851.84	1.64
J-*η*-200-275+	20,414.23	19,992.13	−2.07
J-*η*-200-300+	23,653.88	23,801.61	0.62
J-*η*-150-150+	5941.52	5535.377	−6.84
J-*η*-150-175+	9010.61	8000.551	−11.21
J-*η*-150-200+	11,452.97	10,833.88	−5.41
J-*η*-150-225+	13,742.02	13,558.85	−1.33
J-*η*-150-250+	16,379.9	16,607.65	1.39
J-*γ*-200-6+	15,002.93	15,011.51	0.06
J-*γ*-200-7+	16,023.23	15,632.4	−2.44
J-*γ*-200-8+	16,579.71	16,851.84	1.64
J-*γ*-200-9+	18,122.54	17,820.96	−1.66
J-*γ*-200-10+	19,006.86	19,340.09	1.75
J-*γ*-150-6+	9189.39	9478.365	3.14
J-*γ*-150-7+	9952.77	9824.905	−1.28
J-*γ*-150-8+	11,309.46	10,840.69	−4.14
J-*γ*-150-9+	12,203.30	11,972.49	−1.89
J-*γ*-150-10+	12,361.27	13,326.06	7.80
J-*γ*-200-6+	15,002.93	15,011.51	0.06
J-*τ*-200-4+	12,715.51	12,316.82	−3.14
J-*τ*-200-5+	13,780.39	13,542.24	−1.73
J-*τ*-200-6+	14,287.32	14,236.07	−0.36
J-*τ*-200-7+	14,777.89	14,621.13	−1.06
J-*τ*-200-8+	15,225.99	14,915.25	−2.04
J-*τ*-150-4+	7711.47	8283.747	7.42
J-*τ*-150-5+	8991.67	8738.897	−2.81
J-*τ*-150-6+	9129.09	9123.677	−0.06
J-*τ*-150-7+	9533.8	9403.697	−1.36
J-*τ*-150-8+	9640.53	9530.787	−1.14

## Data Availability

The data presented in this study are available on request from the corresponding author.

## Abbreviations

**Notation**	**Implication**
*η*	Beam height to column flange width ratio
*β^*^*	Beam-column width ratio correction
*γ*	Column tube wall width–thickness ratio
*τ*	Beam-column section wall-thickness ratio
*β*	Beam-column width ratio
*M*_u_	Ultimate bending moment
*t*_l_ *l*	The cross-sectional area of the plate corresponding to the stiffener
*f*_y_	The yield strength of the steel
*K*_0_	The rotational stiffness
Δ*K*_0_	The increment of the initial rotational stiffness
*k*_cw_	The tension-compression deformation stiffness
*P*	The vertical load at the loading point at the beam end
Δ	The vertical displacement at the loading point at the beam end
*h*	Indicates the moment arm provided by the upper and lower stiffeners

## References

[1] SAFRAJE (1999). Encyclopedia Britannica (International Chinese Edition).

[2] (2005). Design of Steel Structures: Design of Joints.

[3] Wang Y., Lei H. (2018). Finite element analysis of static performance and stress concentration of T-shaped circular tube intersecting joints. Chin. Sci. Technol. Pap..

[4] Chen J., Huang H. (2015). In-plane bending stiffness analysis of T-shaped joints in steel tube structures. J. Shijiazhuang Railw. Univ. Nat. Sci. Ed..

[5] Liu J., Guo Y. (2001). Nonlinear finite element analysis of ultimate bearing capacity of intersecting joints of K-shaped square and circular tubes. Build. Sci..

[6] Weng Y., Guan F., Wang X. (2006). Study on Failure Mode and Analysis Model of X-shaped Circular Tube Intersecting Joints. Steel Struct..

[7] Wang L., Wang L. (2008). Ultimate strength analysis of TT-shaped circular tube joints on offshore platforms. Ship Ocean. Eng..

[8] Shu X., Zhu Q. (2002). Finite element analysis of ultimate bearing capacity of intersecting joints of space KK steel tubes. J. South China Univ. Technol. Nat. Sci. Ed..

[9] Packer J.A., Davies G., Coutie M.G. (1982). Ultimate strength of gapped joints in RHS trusses. J. Struct. Eng. ASCE.

[10] Wu Z., Tan H. (2008). Calculation of initial flexural stiffness of T-shaped square steel tube joints with unequal widths. J. Harbin Inst. Technol..

[11] Hectors K., De Waele W. (2020). A numerical framework for determination of stress concentration factor distributions in tubular joints. Int. J. Mech. Sci..

[12] Zhao P., Qian J., Zhao J., Ma H., Gu L. (2005). Research on bearing capacity of intersecting joints of rectangular steel tubes under single stress state. J. Build. Struct..

[13] International Institute of Welding Subcommission XV-E (2012). Static Design Procedure for Welded Hollow Section Joints: Recommendations.

[14] (2013). Static Design Procedure for Welded Hollow Section Joints: Recommendations.

[15] (2010). Technical Specification for Steel Tube Structure.

[16] (2017). Design Standards for Steel Structures.

[17] Yang Y. (2015). Application and Research of Eccentric Tubular Joints.

[18] Zhao B., Ke K., Jiang W. (2018). Research on out-plane flexural performance of unstiffened eccentric RHS joints. J. Huazhong Univ. Sci. Technol. Nat. Sci. Ed..

[19] Zhao B., Jiang W., Ke K., Liu C. (2019). Out-OF-Plane Flexural Rigidity of Unstiffened Eccentric Rectangular Hollow Joints. J. Harbin Inst. Technol..

[20] Guo X., Xu Z., Liu J., Luo J. (2023). In-plane flexural capacity of rectangular steel tube eccentric intersecting beam-column joints. J. Hunan Univ. Nat. Sci. Ed..

[21] Nassiraei H. (2022). Geometrical effects on the LJF of tubular T/Y-joints with doubler plate in offshore wind turbines. Ships Offshore Struct..

[22] Nassiraei H. (2019). Local joint flexibility of CHS X-joints reinforced with collar plates in jacket structures subjected to axial load. Appl. Ocean. Res..

[23] Rao T., Du X., Yuan H., Leng Z., Cao H. (2022). Research on the mechanical performance of intersecting joints of equal-width KX-shaped rectangular steel tubes. Prog. Build. Steel Struct..

